# Characteristics of Mild Cognitive Impairment in Northern Japanese Community-Dwellers from the ORANGE Registry

**DOI:** 10.3390/jcm8111937

**Published:** 2019-11-10

**Authors:** Yu Kume, Tomoko Takahashi, Yuki Itakura, Sangyoon Lee, Hyuma Makizako, Tsuyosi Ono, Hiroyuki Shimada, Hidetaka Ota

**Affiliations:** 1 Department of Occupational Therapy, Graduate School of Medicine, Akita University, Akita 010-8543, Japan; kume.yuu@hs.akita-u.ac.jp; 2 Integrated Community Support Center, Public Health and Welfare Department, City Hall of Yokote, Akita 013-0525, Japan; takahashi-tomoko-d@city.yokote.lg.jp; 3 Advanced Research Center for Geriatric and Gerontology, Akita University, Akita 010-8543, Japan; itacie15@gmail.com; 4 Center for Gerontology and Social Science, National Center for Geriatrics and Gerontology, Obu, Aichi 474-8511, Japan; sylee@ncgg.go.jp (S.L.); shimada@ncgg.go.jp (H.S.); 5 Department of Physical Therapy, School of Health Sciences, Faculty of Medicine, Kagoshima University, Kagoshima 890-8544, Japan; makizako@health.nop.kagoshima-u.ac.jp; 6 Omori Municipal Hospital, Akita 013-0525, Japan; onot@oomorihp.jp

**Keywords:** older adults living in super-aging society, mild cognitive impairment, walking speed, depression

## Abstract

A gradually increasing prevalence of mild cognitive impairment (MCI) is recognized in the super-aging society that Japan faces, and early detection and intervention in community-dwellers with MCI are critical issues to prevent dementia. Although many previous studies have revealed MCI/non-MCI differences in older individuals, information on the prevalence and characteristics of MCI in rural older adults is limited. The aim of this study was to investigate differential characteristics between older adults with and without MCI. The investigation was conducted over one year from 2018 to 2019. Participants were recruited from Akita in northern Japan. Neuropsychological assessments were applied to classify MCI, including the National Center for Geriatrics and Gerontology Functional Assessment Tool (NCGG-FAT) and the Touch panel-type Dementia Assessment Scale (TDAS) based on the Alzheimer’s disease assessment scale. Our samples consisted of 103 older adults divided into 54 non-MCI and 49 MCI. The MCI group had lower scores of all cognitive items. Our results showed that individuals with MCI had significantly slower walking speed (WS) and worse geriatric depression scale (GDS) compared to non-MCI. In addition, WS was significantly associated with some cognitive items in non-MCI, but not in MCI. Finally, we showed that predictive variables of MCI were WS and GDS. Our study provides important information about MCI in rural community-dwellers. We suggest that older adults living in a super-aging society should receive lower limb training, and avoiding depression in older adults through interaction of community-dwellers may contribute to preventing the onset of MCI.

## 1. Introduction

Mild cognitive impairment (MCI) is a transitional state of cognition between normal ageing and dementia that may progress to dementia. MCI is defined by subjective or objective evidence of cognitive decline greater than expected for the individual’s age and education level but that does not interfere notably with activities of daily life, and the early detection and prevention of MCI are a challenge to prevent dementia in older adults [1]. Within established processes for making a diagnosis of MCI [2], some factors in its early detection remain unclear, as well as predictors of reversion from MCI to normal cognition. Differences between individuals without MCI and those with MCI have been studied and reported, which shows that many factors such as a lack of exercise [3], cerebrovascular factors [4], and anxiety [5] affect cognitive function. Especially, it is appreciated that cognitive and physical impairments in older adults are related through shared pathophysiological mechanisms [6]. Some studies show that older adults with MCI compared to individuals without MCI perform more poorly not just on neurocognitive performance, but also on complex motor and psychomotor domains [7,8,9], and exhibit greater gait impairment [10,11,12,13,14]. Recently, it has become clear that MCI and physical frailty are related. The physical phenotype of frailty is represented by low levels of lean body mass, muscle strength, gait performance, physical activity, and exhaustion [15]. Gait performance of the frailty is associated with cognitive decline and MCI conversion to AD as reported by [9,16]. Therefore, investigating close associations between MCI and physical function has important implications for improving diagnostic acuity of MCI and targeting interventions to prevent dementia and disability among older adults.

To clarify the dementia risk associated with MCI or early stage dementia, a nationwide clinical registry called the Organized Registration for the Assessment of dementia on Nationwide General consortium toward Effective treatment (ORANGE) is ongoing in Japan [17]. The recruitment of many registrants has been in progress in several regions of Japan from 2017, and we performed an extending preclinical trial in a cohort in northern Japan up to 2019. As is well known, gradual growth of the older population has been experienced in Japan. Especially, northern rural areas in Japan (Akita prefecture) are the most super-aging society in the world (e.g., the number of individuals over aged 75 in Akita is estimated to reach 205,000 people by 2025 [18]). Although there are few epidemiological data regarding MCI in rural areas of Japan, several studies have reported MCI profiles in older adults [19,20]. Most of them are focused on the prevalence of MCI or the conversion rate to dementia, and the detailed cognitive profile (e.g., attention, executive function, information coding skill, etc.) of MCI is not covered, as well as scarce epidemiological data regarding health-related variables such as physical performance and mental status. Therefore, we analyzed the data of a prospective cohort in northern Japan. In this study, we investigated which factors were related to MCI status according to the National Center for Geriatrics and Gerontology Functional Assessment Tool (NCGG-FAT) [21,22] and the Touch panel-type Dementia Assessment Scale (TDAS) [23,24] based on the Alzheimer’s disease assessment scale (ADAS) [25]. To clarify the characteristics of rural older adults with MCI, we focused on three points as follows. First, we mainly compared cognitive function, physical performance, and depressive symptoms in MCI individuals with those in non-MCI individuals. Second, we examined correlations between physical performance and cognitive and mental function in each group (i.e., non-MCI group and MCI group). Finally, a binomial logistic regression model was estimated to determine predictive factors for MCI in rural older adults in Japan.

## 2. Experimental Section

### 2.1. Participants and Study Design

The participants were recruited in a rural area in Akita with a small population (total 32,440) with a super-aged rate of 38.7% according to public information, from 2018 to 2019. The inclusion criteria were age 65 years and over, having walking ability without personal assistance, and living at home. The exclusion criteria were dementia, major depression, severe hearing or visual impairment, stroke, Parkinson’s disease, other neurological disease, intellectual disability, need for support or care as certified by the Japanese public long-term care insurance system due to disability, and inability to complete cognitive tests at the baseline assessment. The study was approved by the ethics committee of the Faculty of Medicine, Akita University (approval No. 1649) and was performed in accordance with the Declaration of Helsinki II. Informed consent was obtained from all participants. According to sample size calculations using G*Power for unpaired *t* test [26], we estimated a sample size of 64 participants per group to detect a clinically significant effect with α = 0.05, power = 80%, and effect size = 0.50.

### 2.2. Assessment and Outcome

After obtaining informed consent from each participant, demographics (age, gender, and education) and health variables (body mass index (BMI), medical history of hypertension and diabetes, frail phenotype, medication and Geriatric Depression Scale-15 (GDS)) were collected according to the ORANGE protocol. A questionnaire sent in advance by mail was self-described by each participant, including age, gender, educational duration, presence of hypertension and diabetes (e.g., yes or no), amount of medications, and GDS (e.g., score range from 0 to 15, as indexed more depressive symptoms in higher scores). Height and weight to calculate BMI were measured by public health nurses. Five components of the National Center for Geriatrics and Gerontology-Study of Geriatric Syndromes (NCGG-SGS) [27] based on the Fried frailty index [15] were applied to assess frailty: (i) self-reported unintentional weight loss (i.e., a decrease of 2–3 kg over six months [28]), (ii) self-reported exhaustion (i.e., presence of fatigue for two weeks [28]), (iii) self-reported low physical activity (i.e., no exercise habit for a week [29]), (iv) weakness (i.e., grip strength (GS) less than 26/18 kg for male/female [30]): GS was measured using a Smedley-type handheld dynamometer (GRIP-D; Takei Ltd., Niigata, Japan), and (v) slow walking speed (WS) (i.e., less than 1.0 m/s in 5 m walking test [29]): walking time was measured over a 2.4-m distance in seconds using infrared sensors and participants’ WS (m/s) was calculated. They were used to define robust (score of zero), pre-frail (score of 1 to 2), and frail (score of 3 to 5). The frail index of NCGG-SGS is almost equal to the original index of Fried’s study [15] except the modified cut-off values for slowness and weakness are appropriate criteria for physical frailty assessments in the Japanese older population [31,32]. The present study also applied NCGG-FAT and TDAS based on ADAS to assess cognitive function in the participants and to divide the participants into non-MCI and MCI groups. All the variables of five frail components, the NCGG-FAT and TDAS were evaluated by trained public health nurses throughout a comprehensive health checkup in a local spot.

### 2.3. Components of NCGG-FAT

The computerized multidimensional neurocognitive test was performed on an iPad (Apple, Cupertino, CA, USA) with a 9.7-inch touch display. The task instructions were presented with a letter size of at least 1.0 × 1.0 cm^2^ on the display. For this study, a trained operator supported each participant by setting up the tablet PC and running each test. Participants completed the NCGG-FAT subtests as follows.

#### 2.3.1. Tablet Version of Word Recognition (WR)

This test is comprised of two computerized tasks of immediate recognition and delayed recall. In the first task of immediate recognition, participants were instructed to memorize 10 words, each of which was displayed for 2 s on the tablet PC. After that, a total of 30 words including 10 target and 20 distracter words were shown to participants, and they were required to select the 10 target words immediately. This task was repeated for three trials. The average number of correct answers was recorded as a score ranging from 0 to 10. In another task, participants were asked to correctly recall the 10 target words after 20 min. The number of correctly recalled target words was scored ranging from 0 to 10. Finally, we calculated the sum score of the two tasks of immediate recognition and delayed recall.

#### 2.3.2. Tablet Version of Trail Making Test Version A (TMT-A) and Version B (TMT-B)

In the Trail Making Test Version A (TMT-A) task, participants were instructed to touch the target numbers in a sequence as rapidly as possible. Target numbers from 1 to 15 were randomly displayed on the tablet panel. In addition, the Trail Making Test Version B (TMT-B) instructions required participants to touch target numbers (e.g., 1–15) and letters in turn. The required time (seconds) to complete each task was recorded, within a maximum time of 90 s.

#### 2.3.3. Tablet Version of Symbol Digit Substitution Task (SDST)

In the Symbol Digit Substitution Task (SDST), nine pairs of numbers and symbols were shown in the upper part of the tablet display. A target symbol was shown in the center of the tablet panel, and selectable numbers were displayed at the bottom. Participants were asked to touch the number corresponding to the target symbol shown in the central part of the tablet display as rapidly as possible. The number of correct numbers within 90 s was recorded.

### 2.4. Components of TDAS

The TDAS test was presented on a 14-inch touch panel display. The TDAS subtests consisted of seven of the ADAS-cog test items (11 test items) and two other tasks. Participants were instructed verbally or visually by the computer to complete the TDAS subtests as follows.

#### 2.4.1. WR

The WR test was a computerized test based on the WR task of ADAS-cog. At the start of instructions for this task, 12 target words were individually presented on the display for 3 s each at 2 s intervals. After demonstrating the target words, the computer randomly displayed 24 words consisting of 12 target words and 12 non-target words. Participants were then instructed to respond by touching the displayed button of ‘yes’, ‘no’, or ‘unknown’ in response to the question regarding whether the word had been shown previously. Participants completed the trial three times. The total number of incorrect responses for three trials was recorded, with a maximum score of 72.

#### 2.4.2. Following a Command

This task was modified from the command task of ADAS-cog. The computer presented 10 selectable icons labelled from 0 to 9 and then required participants to touch the number specified. The number of incorrect responses in two trials was scored with a maximum score of 2.

#### 2.4.3. Orientation

This task was based on the orientation task of ADAS-cog. The computer displayed four screens in sequence. On each screen, participants were asked to touch selectable icons and answer what year, month, day, and weekday it is. The number of incorrect responses was scored with a maximum score of 4.

#### 2.4.4. Visual-Spatial Perception

This task was modified from the constructional praxis task of ADAS-cog to evaluate visual-spatial perception. ADAS-cog requires subjects to copy the geometric forms presented. The computer first presented four screens displaying a target geometric form (i.e., a square, rhombus, cube, or triangular prism) for 5 s each. Participants were then required to correctly select the target form in response to a question task including the target form and four non-target forms. The number of incorrect responses was scored with a maximum score of 4.

#### 2.4.5. Naming Fingers

This test assessed whether participants can name the fingers correctly, using the protocol of ADAS-cog. Participants were asked to correctly respond to a picture question of a hand marked with a red circle, by touching an icon labelled with the five finger names. An incorrect response was scored as one point, with a maximum score of 5.

#### 2.4.6. Object Recognition

This task was based on the naming objects task of ADAS-cog. Participants were instructed to touch the correct usage icon (e.g., a pair of scissors, comb or broom) of five selectable icons labelled with the purpose of usage. Three trials were completed, and an incorrect response was scored as one point (maximum score = 3).

#### 2.4.7. Accuracy of Order of a Process

This task was modified from the ideational praxis of ADAS-cog. The computer displayed seven icons labelled randomly with seven actions. Participants were asked to correctly touch the icons in order. The number of incorrect responses was recorded, with a maximum score of 5.

#### 2.4.8. Money Calculation

This task assessed the money calculation ability of each participant. Participants needed to combine coins equal to an amount of money from various denominations of coins displayed on the screen. Three trials were completed, and an incorrect response was scored as one point (maximum score = 3).

#### 2.4.9. Clock Time Recognition

This task included three kinds of question regarding clock time recognition. Participants were instructed to correctly state the time shown on a clock displayed on the screen. The number of incorrect responses was recorded, with a maximum score of 3.

### 2.5. MCI Classification by NCGG-FAT and TDAS

According to Petersen’s report [2] in which individuals who showed cognitive impairment but were independent in activities of daily living were defined as having MCI, we applied MCI classification according to the cutoff point of NCGG-FAT or TDAS. For all cognitive subtests of NCGG-FAT, the standardized threshold in each corresponding domain for defining impairment in Japanese population-based cohorts consisting of older community-dwellers is a score more than 1.5 standard deviations (SD) below the age- and education-specific mean [21]. In TDAS, decreasing scores indicate cognitive improvement (range of scores from 0 to 101), and total scores ranging from 7 to 13 were classified as MCI [23].

## 3. Analyses

According to results of the normalization test (Kolmogorov–Smirnov test), Age, Height, Weight, and BMI were used by the unpaired *t* test. Gender (% female), Hypertension (% Yes), Diabetes (% Yes), Weight loss (% Yes), Poor energy (% Yes), and Low physical activity level (% Yes) were analyzed by chi-squared test for 2 × 2 contingency, except for Pearson’s chi-square test for Frail phenotype (%, robust/pre-frail/frail) for 2 × 3 contingency. Mann–Whitney test was applied for GS (kg), WS (m/s), Amount of medications (*n*), Education (years), GDS-15 (score), and cognitive measurements of NCGG-FAT and TDAS (Table 1).

As the variables of WS, GS, subtests of NCGG-FAT, TDAS, and GDS-15 total score were not statistically normalized from the Kolmogorov–Smirnov test, Spearman correlation analysis for interval scales was applied to analyze the relationship among Age, GS, WS, subtests of NCGG-FAT, TDAS, and GDS total score for each group (Table 2).

The values of *p*_in_ = 0.2 and *p*_out_ = 0.25 were set up to select independent variables from Table 1 and Table 2 for input into a binominal logistic regression model. The regression model was performed by a method of likelihood ratio, and set up the MCI classification as the dependent variable and predictors (i.e., independent variables) according to the following regression models; (i) 11 predictors of Model I include Age, GS, WS, Amount of medications, Education, WR, TMT-A, B, and SDST of NCGG-FAT, TDAS, and GDS-15 total score. (ii) Ten predictors of Model II except for TDAS score include Age, GS, WS, Amount of medications, Education, WR, TMT-A, B, and SDST of NCGG-FAT, and GDS-15 total score. Finally, (iii) six predictors of Model III except for all cognitive variables included Age, GS, WS, Amount of medications, Education, and GDS-15 total score. The model adaptation was examined by Hosmer–Lemeshow test (Table 3). SPSS Version 26.0 for Windows (SPSS Inc., Chicago. IL, USA) was used for analysis, and the level of significance was set at *p* = 0.05.

## 4. Results

Our samples consisted of 103 older participants divided into 54 non-MCI people and 49 MCI people. We confirmed that the MCI group had significantly lower scores or longer required times of all cognitive items including WR test, TMT-A, B, SDST and TDAS scores than the non-MCI group (*p* < 0.0001) (Table 1). Demographic and health data including Age, Gender, BMI, presence of Hypertension or Diabetes, Frail phenotype, presence of Weight loss, Poor energy, Low physical activity level, Amount of medications, and Education showed no significant difference between the non-MCI group and MCI group. Of physical assessments, WS was significantly different between the groups (*p* = 0.03), whereas GS was not different (*p* = 0.25). Moreover, the MCI group showed a worse score of GDS (*p* = 0.046 < 0.05). Next, we examined correlations between physical performance, cognitive and mental function in each group (Table 2). According to the results of Spearman correlation analysis, WS was associated with some items of cognitive subtests including WR, TMT-A, B, and SDST in the non-MCI group (|r| > 0.30, *p* < 0.01), but these were not significant in the MCI group except for correlations between cognitive items and Age or Education. Finally, we performed an analysis to determine explanatory variables for MCI with reference to non-MCI by binomial logistic regression analysis (Table 3). According to a result of Phi coefficient of association, all the nominal scales including Gender (Phi coefficient = 0.01, *p* = 0.89), presence of Hypertension (Phi coefficient = 0.02, *p* = 0.86) and Diabetes (Phi coefficient = 0.07, *p* = 0.46), Weight loss (Phi coefficient = 0.05, *p* = 0.63), Poor energy (Phi coefficient = 0.12, *p* = 0.22), Low physical activity level (Phi coefficient = 0.08, *p* = 0.45) were not significantly associated with MCI classification, and they were not included into predictors for the regression model. Three regression models were estimated according to the predictors of Age, GS, WS, Amount of medications, Education, WR, TMT-A, B, SDST, TDAS, and GDS-15 total score. Model I that included them demonstrated that the classification of MCI had a significant association with Age, TMT-B, SDST, and TDAS. Next, Model II except for T-DAS score from Model I was applied to estimate a specific cognitive profile in MCI. Model II demonstrated that the classification of MCI had a significant association with Age, WR, TMT A, B, and GDS-15 total score. Finally, considering the self-explanatory effect of cognitive items, Model III except for all cognitive variables from Model II was applied to clarify the classification of MCI. As shown in Model III, WS and GDS-15 total score were extracted as explanatory variables of MCI (Table S1). In the three estimated models, the results of Hosmer–Lameshow test showed adaptability of 87.4% (*p* = 0.12) in Model I, 84.5% (*p* = 0.84) in Model II, and 59.2% (*p* = 0.02) in Model III.

## 5. Discussion

In this study, we found characteristics of MCI in northern Japanese community-dwellers of super-aging society had slower WS and tendency to depression. Aging continues in the subjects of our survey area, and the population ratio 65 years or older reached 38.7% (July, 2019). Actually, the prevalence of MCI in this study was higher (47.6%) compared with other rural areas which were previously reported to be about 10%–30% [29,33]. Additionally, some wealthy urban areas different from our rural area showed that characteristics of MCI were greater with older age and less education than non-MCI [34,35]. Although this high prevalence and multifactorial approach may be due to different methods, it could also be because our community-dwellers living in an area of heavy snowfall in northern Japan experience a more negative impact on gait performance [36] and a potentially high incidence of depressive symptoms [37] because of fewer opportunities to go out and participate in social activities. In fact, we showed an association between cognitive function and demographic and health data including age, gender, BMI, medical history, medication, frailty phenotype, education, physical performance, and GDS in older adults living in a super-aging society (Table 1). We found that recognized risk factors for MCI including age, gender, BMI, presence of hypertension or diabetes, frailty phenotype, education, and amount of medications were not different, but WS and GDS were significantly different between the groups. We also found that WS was significantly associated with some cognitive items including SDST and TMT in the non-MCI group, but not in the MCI group (Table 2). The regression models demonstrated that MCI had a significant association with age, executive function, information coping speed, and composite cognitive performance, indicating that these are predictive variables for the presence of MCI. However, because of the effect of variables on these cognitive scores (Model I), we applied Models II and III (Table 3). Model II excluding composite cognitive performance, as indexed in the TDAS score, demonstrated that MCI had a significant association with age, WR, attention, executive function, and GDS. Compatible with the results of Reinvang et al. [38], attention and executive dysfunction in neuropsychological tests could be early symptoms of MCI. Especially, the variables of SDST and TMT are recognized to reflect psychomotor processing and executive function [39], and several studies have reported that they are rapidly altered in MCI subjects [40,41]. Although they justify its use for the detection of cognitive impairment in older adults, most of these tests have numerous limitations (the problem of novelty, lack of sensitivity and specificity, patient cognitive reserve, etc.) [42,43]. This recent observation underscores the need to find new detection indicators for cognitive impairment. With this in perspective, a new approach associates WS of older adults with the presence of cognitive impairment.

Interestingly, in Model III excluding all cognitive domains, WS and GDS were selected as explanatory variables although the percentage of correct classifications was not so good in the Hosmer–Lameshow test. These findings indicate that the variables WS and GDS can potentially distinguish the presence or absence of MCI; therefore, they provide suggestive information on the presence of MCI. Recently, some studies have focused on both cognition and locomotor performance as predictors of adverse outcomes in community-dwellers with MCI [44,45]. In particular, slow gait speed at usual pace has been implicated in the onset of adverse outcomes, such as disability [46], cognitive impairment [47], institutionalization, falls [48,49], and mortality [50]. As previously reported, the association between slowing of walking and MCI is supported by shared neurological findings that include a smaller right hippocampus [51]. This finding underscores walking–brain behavior relationships and the value of WS as an early indicator of dementia risk. However, thus far, there is insufficient information to state that WS can potentially predict adverse outcomes in older community-dwellers, and more specific investigations need to be performed. Moreover, we showed that GS was no different between the groups (*p* = 0.25), suggesting that reinforcement of lower, but not upper, limb muscular strength may be a critical target in rehabilitation. Likewise, recent studies have indicated that lower extremity motor dysfunction may be a feature of MCI [52], but little is known about the nature and biological mechanism such as myokines of lower extremity motor dysfunction associated with MCI. Regarding WS and a cognitive function, the concept of frailty has become a geriatric topic recently. Although we could not include frailty as global score in the correlation analysis or binomial regression analysis because the distribution of a frail group according to the frailty phenotype was greatly biased (e.g., % of robust/pre-frail/frail, 50%/50%/0% in the non-MCI group, 43%/47%/10% in the MCI group) (Table 1), some studies have reported that a physical frailty is associated with MCI and a reduction of WS in five items of the Fried index mostly reflect the occurrence of MCI and disability [31,53]. MCI with concomitant physical frailty may be considered to fulfil the criteria for cognitive frailty [54]. In this regard, we believe that the cognitive frailty concept has potential advantages in better stratifying the risk profiles of older adults with MCI. In a comparison between the groups, MCI also showed significantly higher depressive scores as indexed in the GDS. Concerning geriatric depression in MCI, cross-sectional research has shown that the association between depressive symptoms, as indexed in the Korean version of GDS, and memory or executive function was significantly greater in individuals with MCI than in those with AD [55]. Additionally, survival analysis followed for 6.28 years on average, indicating that the presence of MCI is a poor predictive factor in individuals with depressive symptoms as indexed in the GDS [56]. Thus, geriatric depressive symptoms in individuals with MCI need to be carefully screened in rural community-dwellers.

The limitations of our research need to be considered in developing our future research. First, the NCGG-FAT and TDAS used to classify individuals with MCI in this study were a tablet PC version of cognitive measurement tools based on the MCI criteria reported by Petersen [2], and evaluation of the accuracy of MCI’s classification is essential for worldwide research. Second, our cohort was comprised of a localized group of individuals in one rural area of northern Japan, whose actual sample size (*n* = 103) did not reach the calculated required sample size (*n* = 128) due to difficulty sampling and recruiting in a depopulated, small rural area. Third, considering younger age was associated with MCI, we could not take the association into consideration. Fourth, although focusing this study on frailty concept was important, we guessed it was difficult to analyze frail status in detail due to bias of frail samples between the groups (e.g., 0% of the non-MCI group, 10% of the MCI group). Further examination concerning frailty is warranted in future research. Finally, we hypothesize that cognitive domains, gait performance, and tendency to depression might be associated with MCI status. For the three regression models in this study, WS and GDS were selected as explanatory variables in Model III. However, further research with sufficient adaptability should be carried out with a large sample size in multiple rural districts. These limitations need to be considered when interpreting this study’s findings.

## 6. Conclusions

In conclusion, WS and GDS were shown to be potential predictive variables of MCI in our study, and we consider they provide important information about characteristics of MCI in rural community-dwellers. It is suggested that older individuals living in a super-aging society should work on training lower limb muscular strength, and avoiding depression in older adults by interaction of community-dwellers may contribute to prevention of the onset of MCI.

## Figures and Tables

**Table 1:** Characteristics of participants with and without mild cognitive impairment (MCI).

Variables	Non-MCI Group		MCI Group		*p* Value	
	*n* = 54		*n* = 49			
	Mean	SD	Mean	SD		
Age (years)	74.1	6.1	74.4	5.7	0.84	
Gender (% female)	53.7%		55.1%		0.89	
Height (cm)	155.0	8.2	156.0	8.5	0.53	
Weight (kg)	57.9	11.4	60.0	10.1	0.34	
Body Mass Index (kg/m^2^)	24.0	3.7	24.6	3.5	0.41	
	%		%		***p* Value**	
Hypertension (% Yes)	63.0%		61.2%		0.86	
Diabetes (% Yes)	20.4%		26.5%		0.46	
**Frail five components**						
Frail phenotype (%, robust/pre-frail/frail)	50%/50%/0%		43%/47%/10%		0.054	
(i) Weight loss (% Yes)	11.1%		14.3%		0.63	
(ii) Poor energy (% Yes)	16.7%		26.5%		0.22	
(iii) Low physical activity level (% Yes)	13.0%		18.4%		0.45	
	**Median**	**IQR**	**Median**	**IQR**	***p* Value**	
(iv) Grip strength (kg)	25.2	12.0	22.9	9.0	0.25	
(v) Walking speed (m/s)	1.3	0.4	1.2	0.3	0.03	*
Amount of medications (*n*)	3.0	3.0	4.0	4.0	0.17	
Education (years)	12.0	3.0	12.0	3.0	0.18	
GDS-15 total score (score)	2.0	2.0	3.0	4.0	0.046	*
**NCGG-FAT**						
Word recognition (score)	11.7	3.8	8.0	4.2	0.000	***
Tablet version of TMT-A (s)	19.0	6.0	27.0	11.0	0.000	***
Tablet version of TMT-B (s)	33.5	18.0	46.0	44.5	0.000	***
Tablet version of SDST (score)	42.0	12.0	33.0	12.5	0.000	***
**TDAS**						
TDAS total score (score)	2.0	3.0	7.0	9.0	0.000	***

* *p* < 0.05, ** *p* < 0.01, *** *p* < 0.001, Mann–Whitney test was applied for Education (years), Amount of medications (*n*), GDS-15 total score (score), Grip strength (kg), Walking speed (m/s), and cognitive measurements of NCGG-FAT and TDAS. Age, height, weight, and BMI were analyzed by unpaired *t* test, and gender (% female), hypertension (% Yes), diabetes (% Yes), weight loss (% Yes), poor energy (% Yes), and low physical activity level (% Yes) were analyzed by chi-squared test, except for Pearson’s chi-square test for frail phenotype (%, robust/pre-frail/frail). SD, standard deviation; IQR, interquartile range; Loss weight, Loss weight more than 3 kg in six months; TMT-A, Trail Making Test A version; TMT-B, Trail Making Test B version; SDST, Symbol Digit Substitution Task; TDAS, Touch Panel-type Dementia Assessment Scale; GDS-15, Geriatric Depression Scale.

**Table 2:** Correlations for each group (non-MCI and MCI).

Variables	Non-MCI Group (*n* = 54)							MCI Group (*n* = 49)						
	WS	GS	WR	TMT-A	TMT-B	SDST	TDAS	WS	GS	WR	TMT-A	TMT-B	SDST	TDAS
Age (years)	−0.37 **	−0.21	−0.51 **	0.55 **	0.66 **	−0.66 **	0.05	−0.37 *	−0.04	−0.33 *	0.43 **	0.49 **	−0.47 **	0.25
BMI (kg/m^2^)	0.08	0.24	0.18	0.05	0.05	0.02	0.00	−0.19	0.08	0.19	0.01	−0.01	0.03	−0.04
Education (years)	0.19	−0.01	0.03	−0.36 **	−0.35 **	0.15	−0.13	−0.02	0.28	0.09	−0.13	−0.10	0.29	−0.45 **
Medications (*n*)	0.06	−0.22	−0.11	−0.02	0.22	0.01	−0.16	−0.24	0.25	0.04	−0.03	0.07	−0.07	0.06
WS (m/s)	1.00	0.26	0.42 **	−0.31 **	−0.35 **	0.44 **	−0.05	1.00	−0.08	−0.08	−0.18	−0.07	0.12	−0.06
GS (kg)	0.26	1.00	0.18	−0.09	−0.02	0.22	0.31 *	−0.08	1.00	0.06	0.16	−0.12	0.16	−0.11
GDS-15 (score)	0.16	0.01	0.03	0.00	−0.01	−0.04	0.00	0.03	−0.16	0.12	−0.13	−0.28	0.12	−0.14

* *p* < 0.05, ** *p* < 0.01, Statistics represent Spearman *r* correlations for each parameter. BMI, body mass index; WS, walking speed; GS, grip strength; WR, word recognition; TMT-A, Trail Making Test A version; TMT-B, Trail Making Test B version; SDST, Symbol Digit Substitution Task; TDAS, Touch Panel-type Dementia Assessment Scale; GDS-15, Geriatric Depression Scale-15.

**Table 3:** Multiple comparison among binomial logistic regression models depending on MCI classification with odds ratio.

Model	Coefficient (β)	Odds Ratio	95% CI	*p* Value
**Model I**				
Age (years)	−0.27	0.77	0.66, 0.89	0.000
TMT-B (s)	0.08	1.09	1.04, 1.14	0.001
SDST (score)	−0.12	0.88	0.80, 0.97	0.012
TDAS total score (score)	0.65	1.91	1.37, 2.68	0.000
**Model II**				
Age (years)	−0.26	0.77	0.67, 0.88	0.000
WR (score)	−0.55	0.58	0.44, 0.76	0.000
TMT-A (s)	0.17	1.19	1.07, 1.33	0.001
TMT-B (s)	0.05	1.05	1.01, 1.10	0.025
GDS-15 total score (score)	0.32	1.37	1.07, 1.77	0.014
**Model III**				
Walking speed (m/s)	−2.29	0.10	0.02, 0.69	0.020
GDS-15 total score (score)	0.20	1.22	1.04, 1.43	0.015

Reference group for analysis was non-MCI group. Model I: Model χ^2^ test, *p* < 0.0001; The Hosmer–Lemeshow test, *p* = 0.12; Percentage of correct classifications = 87.4%. Model II: Model χ^2^ test, *p* < 0.0001; The Hosmer–Lemeshow test, *p* = 0.84; Percentage of correct classifications = 84.5%. Model III: Model χ^2^ test, *p* = 0.002; The Hosmer–Lemeshow test, *p* = 0.02; Percentage of correct classifications = 59.2%. CI, confidence interval; WR, word recognition; TMT-A, Trail Making Test A version; TMT-B, Trail Making Test B version; SDST, Symbol Digit Substitution Task; TDAS, Touch Panel-type Dementia Assessment Scale; GDS, Geriatric Depression Scale-15.

## Supplementary Materials

The following are available online at https://www.mdpi.com/2077-0383/8/11/1937/s1, Table S1: Methodology of the binomial logistic regression models.

## References

[1] Petersen R.C., Stevens J.C., Ganguli M., Tangalos E.G., Cummings J.L., DeKosky S.T. (2001). Practice parameter: Early detection of dementia: Mild cognitive impairment (an evidence-based review). Report of the Quality Standards Subcommittee of the American Academy of Neurology. Neurology.

[2] Petersen R.C. (2004). Mild cognitive impairment as a diagnostic entity. J. Intern. Med..

[3] Öhman H., Savikko N., Strandberg T.E., Pitkälä K.H. (2014). Effect of physical exercise on cognitive performance in older adults with mild cognitive impairment or dementia: A systematic review. Dement. Geriatr. Cogn. Disord..

[4] McKetton L., Cohn M., Tang-Wai D.F., Sobczyk O., Duffin J., Holmes K.R., Poublanc J., Sam K., Crawley A.P., Venkatraghavan L. (2019). Cerebrovascular resistance in healthy aging and mild cognitive impairment. Front. Aging Neurosci..

[5] Mirza S.S., Ikram M.A., Bos D., Mihaescu R., Hofman A., Tiemeier H. (2017). Mild cognitive impairment and risk of depression and anxiety: A population-based study. Alzheimers Dement..

[6] Panza F., Seripa D., Solfrizzi V., Tortelli R., Greco A., Pilotto A., Logroscino G. (2015). Targeting cognitive frailty: Clinical and neurobiological roadmap for a single complex phenotype. J. Alzheimers Dis..

[7] Kluger A., Gianutsos J.G., Golomb J., Ferris S.H., George A.E., Franssen E., Reisberg B. (1997). Patterns of motor impairment in normal aging, mild cognitive decline, and early Alzheimer’s disease. J. Gerontol. B Psychol. Sci. Soc. Sci..

[8] McGough E.L., Kelly V.E., Logsdon R.G., McCurry S.M., Cochrane B.B., Engel J.M., Teri L. (2011). Associations between physical performance and executive function in older adults with mild cognitive impairment: Gait speed and the timed “up & go” test. Phys. Ther..

[9] Aggarwal N.T., Wilson R.S., Beck T.L., Bienias J.L., Bennett D.A. (2006). Motor dysfunction in mild cognitive impairment and the risk of incident Alzheimer disease. Arch. Neurol..

[10] McGough E.L., Cochrane B.B., Pike K.C., Logsdon R.G., McCurry S.M., Teri L. (2013). Dimensions of physical frailty and cognitive function in older adults with amnestic mild cognitive impairment. Ann. Phys. Rehabil. Med..

[11] Fitzpatrick A.L., Buchanan C.K., Nahin R.L., DeKosky S.T., Atkinson H.H., Carlson M.C., Williamson J.D. (2007). Associations of gait speed and other measures of physical function with cognition in a healthy cohort of elderly persons. J. Gerontol. A Biol. Sci. Med. Sci..

[12] Montero-Odasso M., Muir S.W., Speechley M. (2012). Dual-task complexity affects gait in people with mild cognitive impairment: The interplay between gait variability, dual tasking, and risk of falls. Arch. Phys. Med. Rehabil..

[13] Doi T., Makizako H., Shimada H., Yoshida D., Ito K., Kato T., Ando H., Suzuki T. (2012). Brain atrophy and trunk stability during dual-task walking among older adults. J. Gerontol. A Biol. Sci. Med. Sci..

[14] Hooghiemstra A.M., Ramakers I.H.G.B., Sistermans N., Pijnenburg Y.A.L., Aalten P., Hamel R.E.G., Melis R.J., Verhey F.R., Olde Rikkert M.G., Scheltens P. (2017). Gait speed and grip strength reflect cognitive impairment and are modestly related to incident cognitive decline in memory clinic patients with subjective cognitive decline and mild cognitive impairment: Findings from the 4C Study. J. Gerontol. A Biol. Sci. Med. Sci..

[15] Fried L.P., Tangen C.M., Walston J., Newman A.B., Hirsch C., Gottdiener J., Seeman T., Tracy R., Kop W.J., Burke G. (2001). Cardiovascular Health Study Collaborative Research G. Frailty in older adults: Evidence for a phenotype. J. Gerontol. A Biol. Sci. Med. Sci..

[16] Buchman A.S., Boyle P.A., Wilson R.S., Tang Y., Bennett D.A. (2007). Frailty is associated with incident Alzheimer’s disease and cognitive decline in the elderly. Psychosom. Med..

[17] Saji N., Sakurai T., Suzuki K., Mizusawa H., Toba K. (2016). ORANGE investigators. ORANGE’s challenge: Developing wide-ranging dementia research in Japan. Lancet Neurol..

[18] Japanese Ministry of Health, Labour and Welfare (2016). Health and Welfare Bureau for the Elderly. Long-Term Care Insurance System of Japan. https://www.mhlw.go.jp/english/policy/care-welfare/care-welfare-elderly/dl/ltcisj_e.pdf.

[19] Wada-Isoe K., Uemura Y., Nakashita S., Yamawaki M., Tanaka K., Yamamoto M., Shimokata H., Nakashima K. (2012). Prevalence of dementia and mild cognitive impairment in the rural island Town of Ama-cho, Japan. Dement. Geriatr. Cogn. Dis. Extra.

[20] Ishikawa T., Ikeda M., Matsumoto N., Shigenobu K., Brayne C., Tanabe H. (2006). A longitudinal study regarding conversion from mild memory impairment to dementia in a Japanese community. Int. J. Geriatr. Psychiatry.

[21] Makizako H., Shimada H., Park H., Doi T., Yoshida D., Uemura K., Tsutsumimoto K., Suzuki T. (2013). Evaluation of multidimensional neurocognitive function using a tablet personal computer: Test-retest reliability and validity in community-dwelling older adults. Geriatr. Gerontol. Int..

[22] Shimada H., Makizako H., Doi T., Lee S., Lee S. (2017). Conversion and reversion rates in Japanese older people with mild cognitive impairment. J. Am. Med. Dir. Assoc..

[23] Inoue M., Jinbo D., Nakamura Y., Taniguchi M., Urakami K. (2009). Development and evaluation of a computerized test battery for Alzheimer’s disease screening in community-based settings. Am. J. Alzheimers Dis. Other Dement..

[24] Inoue M., Jimbo D., Taniguchi M., Urakami K. (2011). Touch Panel-type Dementia Assessment Scale: A new computer-based rating scale for Alzheimer’s disease. Psychogeriatrics.

[25] Mohs R.C., Cohen L. (1988). Alzheimer’s Disease Assessment Scale (ADAS). Psychopharmacol. Bull..

[26] Faul F., Erdfelder E., Buchner A., Lang A.G. (2009). Statistical power analyses using G*Power 3.1: Tests for correlation and regression analyses. Behav. Res. Methods.

[27] Shimada H., Makizako H., Lee S., Doi T., Lee S., Tsutsumimoto K., Harada K., Hotta R., Bae S., Nakakubo S. (2016). Impact of cognitive frailty on daily activities in older persons. J. Nutr. Health Aging.

[28] Fukutomi E., Okumiya K., Wada T., Sakamoto R., Ishimoto Y., Kimura Y., Chen W.L., Imai H., Kasahara Y., Fujisawa M. (2015). Relationships between each category of 25-item frailty risk assessment (Kihon Checklist) and newly certified older adults under Long-Term Care Insurance: A 24-month follow-up study in a rural community in Japan. Geriatr. Gerontol. Int..

[29] Shimada H., Makizako H., Doi T., Yoshida D., Tsutsumimoto K., Anan Y., Uemura K., Ito T., Lee S., Park H. (2013). Combined Prevalence of Frailty and Mild Cognitive Impairment in a Population of Elderly Japanese People. J. Am. Med. Dir. Assoc..

[30] Chen L.K., Liu L.K., Woo J., Assantachai P., Auyeung T.W., Bahyah K.S., Chou M.Y., Chen L.Y., Hsu P.S., Krairit O. (2014). Sarcopenia in Asia: Consensus Report of the Asian Working Group for Sarcopenia. J. Am. Med. Dir. Assoc..

[31] Makizako H., Shimada H., Doi T., Tsutsumimoto K., Suzuki T. (2015). Impact of physical frailty on disability in community-dwelling older adults: A prospective cohort study. BMJ Open.

[32] Tsutsumimoto K., Doi T., Makizako H., Hotta R., Nakakubo S., Makino K., Suzuki T., Shimada H. (2017). The association between anorexia of aging and physical frailty: Results from the national center for geriatrics and gerontology’s study of geriatric syndromes. Maturitas.

[33] Shimada H., Makizako H., Doi T., Tsutsumimoto K., Lee S., Suzuki T. (2016). Cognitive impairment and disability in older Japanese adults. PLoS ONE.

[34] Kluger A., Gianutsos J.G., Golomb J., Wagner A., Wagner D., Scheurich S. (2008). Clinical features of MCI: Motor changes. Int. Psychogeriatr..

[35] Ravaglia G., Forti P., Montesi F., Lucicesare A., Pisacane N., Rietti E., Dalmonte E., Bianchin M., Mecocci P. (2008). Mild cognitive impairment: Epidemiology and dementia risk in an elderly Italian population. J. Am. Geriatr. Soc..

[36] Mizumoto A., Ihira H., Makino K., Saitoh S., Ohnishi H., Furuna T. (2015). Physical activity changes in the winter in older persons living in northern Japan: A prospective study. BMC Geriatr..

[37] Lewy A.J., Emens J.S., Songer J.B., Sims N., Laurie A.L., Fiala S.C., Buti A.L. (2009). Winter depression: Integrating mood, circadian rhythms, and the sleep/wake and light/dark cycles into a bio-psycho-social-environmental model. Sleep Med. Clin..

[38] Reinvang I., Grambaite R., Espeseth T. (2012). Executive dysfunction in MCI: Subtype or early symptom. Int. J. Alzheimers Dis..

[39] Lowry K.A., Brach J.S., Nebes R.D., Studenski S.A., VanSwearingen J.M. (2012). Contributions of cognitive function to straight- and curved-path walking in older adults. Arch. Phys. Med. Rehabil..

[40] Chapman R.M., Mapstone M., McCrary J.W., Gardner M.N., Porsteinsson A., Sandoval T.C., Guillily M.D., DeGrush E., Reilly L.A. (2011). Predicting conversion from mild cognitive impairment to Alzheimer’s disease using neuropsychological tests and multivariate methods. J. Clin. Exp. Neuropsychol..

[41] Clark L.R., Schiehser D.M., Weissberger G.H., Salmon D.P., Delis D.C., Bondi M.W. (2012). Specific measures of executive function predict cognitive decline in older adults. J. Int. Neuropsychol. Soc..

[42] Brooks B.L., Strauss E., Sherman E.M.S., Iverson G.L., Slick D.J. (2009). Developments in neuropsychological assessment: Refining psychometric and clinical interpretive methods. Can. Psychol..

[43] Weston A.L., Weinstein A.M., Barton C., Yaffe K. (2010). Potentially inappropriate medication use in older adults with mild cognitive impairment. J. Gerontol. A Biol. Sci. Med. Sci..

[44] Perrochon A., Kemoun G. (2014). The Walking Trail-Making Test is an early detection tool for mild cognitive impairment. Clin. Interv. Aging.

[45] Mielke M.M., Roberts R.O., Savica R., Cha R., Drubach D.I., Christianson T., Pankratz V.S., Geda Y.E., Machulda M.M., Ivnik R.J. (2013). Assessing the temporal relationship between cognition and gait: Slow gait predicts cognitive decline in the Mayo Clinic Study of Aging. J. Gerontol. A Biol. Sci. Med. Sci..

[46] Abe T., Kitamura A., Taniguchi Y., Amano H., Seino S., Yokoyama Y., Nishi M., Narita M., Ikeuchi T., Fujiwara Y. (2019). Pathway from gait speed to incidence of disability and mortality in older adults: A mediating role of physical activity. Maturitas.

[47] Abellan van Kan G., Rolland Y., Andrieu S., Bauer J., Beauchet O., Bonnefoy M., Cesari M., Donini L.M., Gillette Guyonnet S., Inzitari M. (2009). Gait speed at usual pace as a predictor of adverse outcomes in community-dwelling older people an International Academy on Nutrition and Aging (IANA) Task Force. J. Nutr. Health Aging.

[48] Kyrdalen I.L., Thingstad P., Sandvik L., Ormstad H. (2019). Associations between gait speed and well-known fall risk factors among community-dwelling older adults. Physiother. Res. Int..

[49] Nakakubo S., Doi T., Makizako H., Tsutsumimoto K., Hotta R., Kurita S., Kim M., Suzuki T., Shimada H. (2018). Association of walk ratio during normal gait speed and fall in community-dwelling elderly people. Gait Posture.

[50] Veronese N., Stubbs B., Volpato S., Zuliani G., Maggi S., Cesari M., Lipnicki D.M., Smith L., Schofield P., Firth J. (2018). Association between gait speed with mortality, cardiovascular disease and cancer: A systematic review and meta-analysis of prospective cohort studies. J. Am. Med. Dir. Assoc..

[51] Rosso A.L., Verghese J., Metti A.L., Boudreau R.M., Aizenstein H.J., Kritchevsky S., Harris T., Yaffe K., Satterfield S., Studenski S. (2017). Slowing gait and risk for cognitive impairment: The hippocampus as a shared neural substrate. Neurology.

[52] Boyle P.A., Wilson R.S., Buchman A.S., Aggarwal N.T., Tang Y., Arvanitakis Z., Kelly J., Bennett D.A. (2007). Lower extremity motor function and disability in mild cognitive impairment. Exp. Aging Res..

[53] Park E.Y., Park S.M., Kim J.H. (2019). Psychometric properties of the geriatric quality of life-dementia in older adults with dementia or mild cognitive impairment living in nursing homes. BMC Geriatr..

[54] Kelaiditi E., Cesari M., Canevelli M., Van Kan G.A., Ousset P.J., Gillette-Guyonnet S., Ritz P., Duveau F., Soto M.E., Provencher V. (2013). Cognitive frailty: Rational and definition from an (IANA/IAGG) international consensus group. J. Nutr. Health Aging.

[55] Lee C.H., Kim D.H., Moon Y.S. (2019). Differential associations between depression and cognitive function in MCI and AD: A cross-sectional study. Int. Psychogeriatr..

[56] Steffens D.C., McQuoid D.R., Potter G.G. (2014). Amnestic mild cognitive impairment and incident dementia and Alzheimer’s disease in geriatric depression. Int. Psychogeriatr..

