# Supplementary figures and images for: Characteristics of Mild Cognitive Impairment in Northern Japanese Community-Dwellers from the ORANGE Registry
Source: J Clin Med. 2019 Nov 10;8(11):1937. doi: 10.3390/jcm8111937 (PMC6912714; doi:10.3390/jcm8111937)

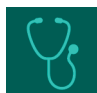

**Table S1.** Methodology of the binomial logistic regression models.

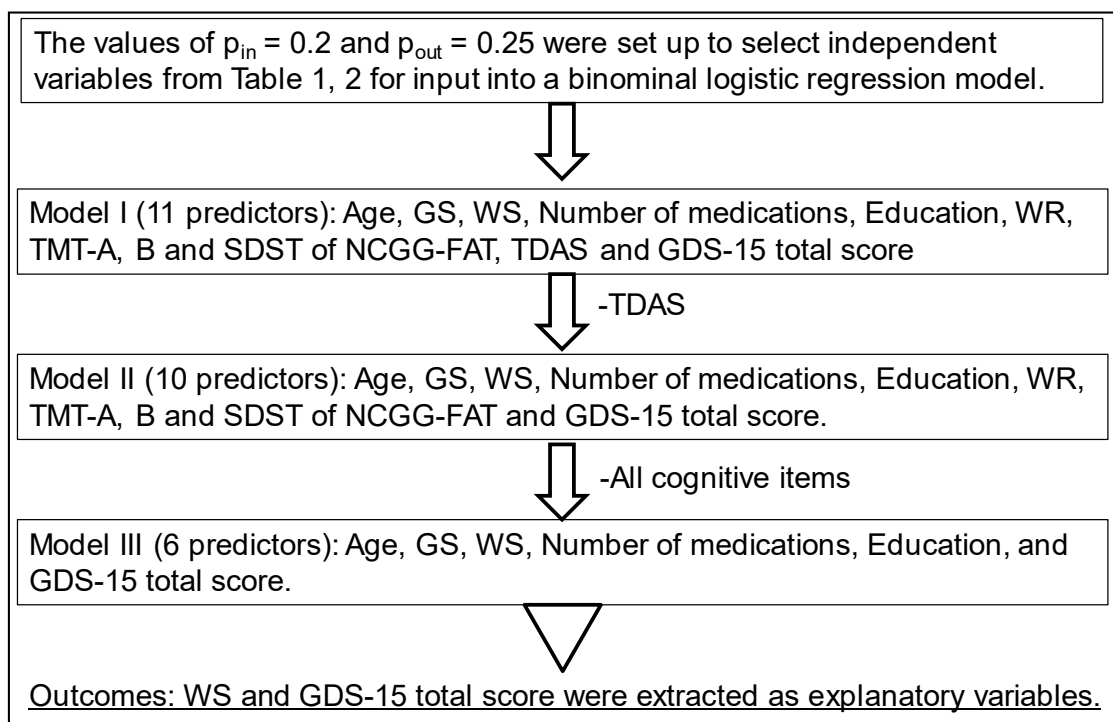

Supplement: Supplementary file 1 [file jcm-08-01937-s001.pdf]
